# Antibacterial activity of ibezapolstat against antimicrobial-resistant clinical strains of *Clostridioides difficile*

**DOI:** 10.1128/aac.01621-23

**Published:** 2024-02-16

**Authors:** Eugénie Bassères, Taryn A. Eubank, Khurshida Begum, M. Jahangir Alam, Jinhee Jo, Thanh M. Le, Chris K. Lancaster, Anne J. Gonzales-Luna, Kevin W. Garey

**Affiliations:** 1 Department of Pharmacy Practice and Translational Research, University of Houston College of Pharmacy, Houston, Texas, USA; University of Pittsburgh, Pittsburgh, Pennsylvania, USA

**Keywords:** *Clostridioides difficile *infection, time-kill studies, bactericidal effects, multidrug-resistant organisms

## Abstract

Antimicrobial resistance is emerging in clinical strains of *Clostridioides difficile*. Ibezapolstat (IBZ) is a DNA polymerase IIIC inhibitor that has completed phase II clinical trials. IBZ has potent *in vitro* activity against wild-type, susceptible strains but its effect on *C. difficile* strains with reduced susceptibility to metronidazole (MTZ), vancomycin (VAN), or fidaxomicin (FDX) has not been tested. The primary objective of this study was to test the antibacterial properties of IBZ against multidrug-resistant *C. difficile* strains. The *in vitro* activity, bactericidal, and time-kill activity of IBZ versus comparators were evaluated against 100 clinical strains of which 59 had reduced susceptibility to other *C. difficile* antibiotics. Morphologic changes against a multidrug resistance strain were visualized by light and scanning electron microscopy. The overall IBZ MIC_50/90_ values (µg/mL) for evaluated *C. difficile* strains were 4/8, compared with 2/4 for VAN, 0.5/1 for FDX, and 0.25/4 for MTZ. IBZ MIC_50/90_ values did not differ based on non-susceptibility to antibiotic class or number of classes to which strains were non-susceptible. IBZ bactericidal activity was similar to the minimum inhibitory concentration (MIC) and maintained in wild-type and non-susceptible strains. Time-kill assays against two laboratory wild-type and two clinical non-susceptible strains demonstrated sustained IBZ activity despite reduced killing by comparator antibiotics for IBZ and VAN non-susceptible strains. Microscopy visualized increased cell lengthening and cellular damage in multidrug-resistant strains exposed to IBZ sub-MIC concentrations. This study demonstrated the potent antibacterial activity of IBZ against a large collection of *C. difficile* strains including multidrug-resistant strains. This study highlights the therapeutic potential of IBZ against multidrug-resistant strains of *C. difficile*.

## INTRODUCTION

*Clostridioides difficile* is a Gram-positive, toxin- and spore-forming obligate anaerobe and the most common cause of infectious diarrhea in hospitalized patients (1). Only two antibiotics, vancomycin (VAN) and fidaxomicin (FDX), are guidelines recommended for *C. difficile* infection (CDI) (2), and antimicrobial resistance has been reported to both (3, 4). A third antibiotic, metronidazole (MTZ), is no longer a guideline recommended due to decreasing clinical response rates most likely due to antimicrobial resistance (5, 6). Although novel therapeutics are in development for CDI, including the recent approval of live biotherapeutic products, only three antibiotics have made it to phase III trials since the approval of FDX in 2011, and none have filed for Food and Drug Administration approval (7–12). Thus, new antibiotics and particularly those with activity against multidrug-resistant (MDR) strains are urgently needed.

Ibezapolstat (IBZ) is a DNA polymerase IIIC (polIIIC) inhibitor that has recently completed phase II clinical trials (13, 14). PolIIIC is a distinct site of action compared to those targeted by currently available antimicrobials. The DNA polIIIC enzyme is essential for the replication of low G + C content of Gram-positive bacteria and is selective for species within the Bacillota phylum (15). As a unique target of the replisome, IBZ may have activity against *C. difficile* strains with resistance to other antimicrobial classes. However, the activity of IBZ on MDR *C. difficile* strains has not been studied. Therefore, this project’s purpose was to determine the efficacy of IBZ against *C. difficile* strains with various patterns of antimicrobial resistance.

## MATERIALS AND METHODS

### Strains and ribotyping

Clinical *C. difficile* strains (*n* = 100) comprising the most common PCR ribotypes (RT) in Texas were selected from our ongoing Texas Surveillance System (16) and included along with laboratory strains R20291 (RT027) and CD630 (RT012) for minimum inhibitory concentration (MIC), bactericidal testing, and microscopy. MDR strains were selected from our collection to fulfill the study objectives. Fluorescent PCR ribotyping was done as previously described (17). The surveillance study from which clinical strains were selected is approved by the University of Houston Committee for the Protection of Human Subjects (CPHS00128).

### Antibiotics

IBZ powder was provided by the study sponsor (Acurx Pharmaceuticals, Inc., Staten Island, NY, USA). FDX and VAN were purchased from Sigma-Aldrich, Inc. (St. Louis, MO, USA). For all assays, antibiotics were diluted in dimethyl sulfoxide (IBZ and FDX) or diluted water (VAN, MTZ) and further diluted with distilled water to reach the final desired concentration. Non-susceptibility to each antibiotic agent was assessed using agar dilution MIC values. Determination of VAN (MIC > 2 mg/L) and FDX (MIC > 1 mg/L) non-susceptibility was based on epidemiologic cutoff values (18–20).

### Agar dilution MIC testing

Isolates were streaked onto a blood agar plate and incubated overnight. A single isolated colony from the blood agar plate was then suspended in brain heart infusion (BHI) broth to achieve turbidity equal to the 0.5 McFarland standard. Agar dilution MIC testing was performed in compliance with Clinical and Laboratory Standards Institute (CLSI) guidance for anaerobic bacteria and as previously described (6, 19). Briefly, the *C. difficile* suspension at a final concentration of ~10^5-6^ colony-forming units (CFU)/m was spotted on Brucella agar (Criterion Media) plates supplemented with hemin (5 µg/mL) (Sigma), vitamin K (10 µg/mL) (Sigma), defibrinated sheep blood (5% vol/vol) (Northeast Lab Services), and doubling the dilution of antibiotics (IBZ, VAN, FDX, and MTZ) from 0.25 mg/L to 16 mg/L. All assays were performed at least in duplicate. MIC assays were repeated for any results with discordant results (>1 dilution difference) within duplicate runs. Isolates out of range were repeated with dilutions up to 64 µg/mL. Reference strain *C. difficile* R20291 was included as a control strain within each experimental run as opposed to the CLSI control strain ATCC 70057.

### ATP-bioluminescence assay

The BacTiter-Glo assay (Promega Corp., Madison, WI, USA) was used to assess IBZ bactericidal activity on the 100 clinical strains stratified by antibiotic susceptibility. Methods were adapted from the manufacturer instructions based on the publication by Jarrad et al. (21). Total unfiltered relative luminescence units were measured with a microplate reader and normalized to the positive growth control (no antibiotic) to account for signal decays according to the formula: % Relative luminescence = (well luminescence − mean of media background)/(mean of growth control − mean of media background) × 100%.

### Time-kill kinetic studies

Two laboratory strains (R20291 and CD630) and two clinical strains [MT5094, a VAN non-susceptible (MIC = 16) strain and FDXR28, an FDX non-susceptible (MIC > 16) strain] were used to test bactericidal effect of wild-type versus non-susceptible strains for IBZ and comparators. *C. difficile* suspension was added to microtiter plates along with fixed concentrations (4–64 µg/mL) of IBZ or comparators (VAN, FDX). Total viable counts were determined immediately (T0; control) and at 24-h post-inoculation. Samples were withdrawn at each time point, centrifuged (1 min at 16 ,000 × *g*), and washed twice in sterile pre-reduced phosphate buffered saline (Oxoid Ltd, Waltham, MA, USA) to reduce residual drug carryover, before 10-fold serial dilutions were performed prior to plating on BHIS agar supplemented with yeast extract and L-cysteine. Agar plates were incubated for 24 h, following which the number of viable *C. difficile* (CFU/mL) was determined. The limit of detection for killing kinetic assays was 200 CFU/mL. Bactericidal activity was defined as a reduction of ≥3log_10_ in viability relative to the starting inoculum after 24-h exposure to antibiotics. Bacteriostatic was defined as <3log_10_ killing compared to the starting inoculum.

### Light microscopy

Light microscopy was done to visualize morphologic differences upon IBZ exposure to a reference strain (R20291: RT027) and clinical strains with reduced susceptibility to VAN (MT4883: RT027) and FDX (FDXR28; RT255). Samples (5 mL) from time-kill studies performed with IBZ 0.5 MIC and 24 h of incubation were centrifuged for 1 min at 10,000 rpm. The pellets were resuspended in 200 µL of 4% paraformaldehyde and incubated for 1 h at room temperature. The samples were centrifuged again for 1 min at 10,000 rpm, resuspended in 1 mL H_2_O, and stored at 4°C prior to microscopy experiments. Light microscopy was performed using an inverted light microscope (Evos Cell Imaging System; Thermo Fisher).

### Experimental plan and analysis

Summary values were calculated and tabulated for all MIC (MIC_50_ and MIC_90_) and ATP (ATP_50_ and ATP_90_) assays. MIC differences between antibiotics were assessed using analysis of variance (ANOVA). Log_10_ CFU/mL reduction from time-kill curves were graphed, tabulated, and compared between antibiotics using the Kruskal Wallis ANOVA. Data visualization and analysis were done with SAS version 9.1 (SAS Institute, Cary, NC, USA) and RStudio (RStudio package version 2023.09.1+494).

## RESULTS

### Activity of ibezapolstat against antibiotic non-susceptible *C. difficile* strains

One hundred clinical isolates were tested to assess the antibacterial of IBZ against multidrug-resistant strains. Isolates were collected between 2017 and 2018 in Texas, USA. The most common ribotypes included were F027, F014-020, F002, and F106. The strains included 41 antibiotic-susceptible strains, 39 VAN non-susceptible strains, 39 FDX non-susceptible strains, and 34 MTZ non-susceptible strains. Many (*n* = 33) non-susceptible strains displayed reduced susceptibility to more than one antibiotic. The activity of IBZ and comparator antibiotics is shown for duplicate independent inocula in Table 1. The overall IBZ MIC_50/90_ values (µg/mL) for IBZ for evaluated *C. difficile* strains were 4/8, compared with 2/4 for VAN, 0.5/1 for FDX, and 0.25/4 for MTZ. Results from the ANOVA demonstrated that all MIC determinations were statistically different from each other (*P* < 0.01 for each comparison) except for FDX and MTZ which had similar MIC distribution. IBZ MIC_50/90_ values did not differ based on non-susceptible antibiotic class or number of classes to which strains were non-susceptible. IBZ bactericidal activity was like the MIC and maintained in wild-type and non-susceptible strains. The overall IBZ ATP_50/90_ values for IBZ for evaluated *C. difficile* strains were 4/16 with no differences observed between ribotype 027 (4/8) compared to other ribotype strains.

**TABLE 1:** Ibezapolstat agar dilution and ATP bactericidal activity against multidrug-resistant *Clostridioides difficile* strains

		Ibezapolstat			
	*N*	MIC_50_	MIC_90_	ATP_50_	ATP_90_
Ribotype					
All	100	4	8	4	16
F027	27	4	8	4	8
F106	13	8	16	4	32
F014-020	12	4	8	4	8
F002	7	8	8	4	16
Other	41	4	8	4	32
Susceptibility					
VAN susceptible	61	8	8	4	32
VAN non-susceptible	39	4	8	4	8
FDX susceptible	61	4	8	4	16
FDX non-susceptible	39	4	8	4	32
MTZ susceptible	66	8	8	4	32
MTZ non-susceptible	34	4	8	4	8
No. of antibiotics^*a*^ non-susceptible					
0	41	4	8	4	32
1	26	8	8	4	32
2	13	4	8	4	8
3	20	4	4	2	8

^*a*^ Antibiotics: VAN, FDX, and MTZ.

### Time-kill studies

**Fig 1:**
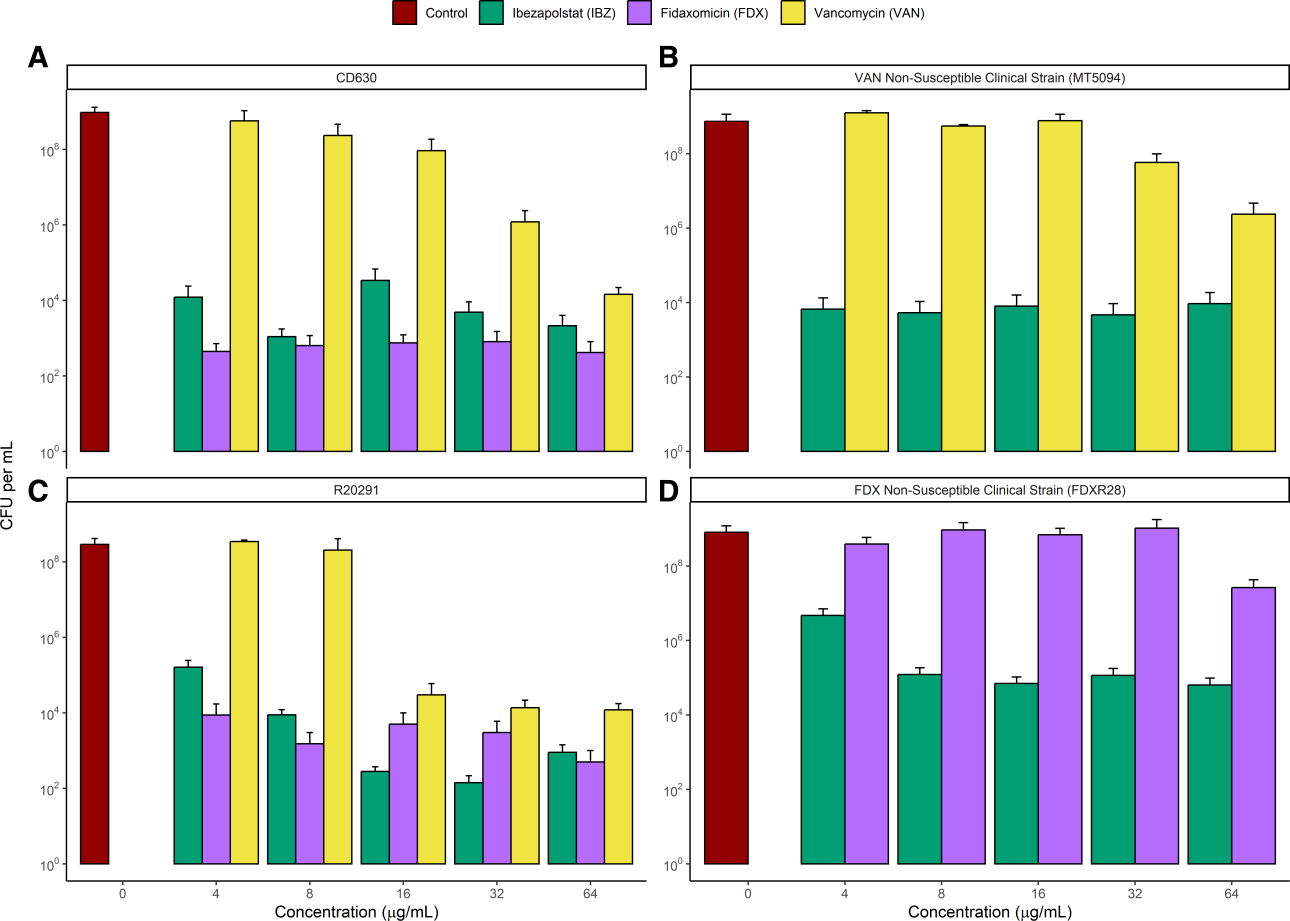
Time-kill results for IBZ, FDX, and VAN against *C. difficile* laboratory strains CD630 (panel A), R20291 (panel C), MT5034, a VAN non-susceptible strain (panel B), and FDXR28, a FDX non-susceptible strain (panel D). Limit of detection, 50 CFU/mL. Error bars represent standard error.

The results of time-kill studies at 24-h evaluation for IBZ, FDX, and VAN against CD630, R20291, MT5094, a VAN non-susceptible isolate, and FDXR28, an FDX non-susceptible isolate are shown in Fig. 1. The corresponding changes in log_10_ CFU/mL relative to baseline are shown in Table 2. All antibiotics exhibited bacteriostatic activity against wild-type, laboratory strains. For laboratory strains (R20291 and CD630), FDX bactericidal activity for both strains (>3log_10_ change) at all concentrations except for higher concentrations (32–64 µg/mL) for R20291, an RT027 strain. For IBZ, >2log_10_ change was observed for both strains except for lower concentrations (4–8 µg/mL) for RT027. VAN achieved >2log_10_ killing for CD630 only at the highest concentration tested (64 µg/mL) but consistently achieved >2log_10_ killing for R20291 except for one concentration (8 µg/mL). Results from the ANOVA demonstrated that all antibiotics were superior to control for wild-type, laboratory strains (*P* < 0.0001). For CD630, the killing activity of FDX and IBZ was significantly better than VAN while for R20291, the killing activity of FDX was significantly better than IBZ which was significantly better than VAN. IBZ maintained at least bacteriostatic concentrations for FDX and VAN non-susceptible strains which was not maintained by FDX and VAN. IBZ log_10_ killing ranged from 0.68 to 1.9 relative to baseline for the FDXR28 while FDX log_10_ killing showed increased growth (1.26–2.64log_10_ increase). IBZ log_10_ killing was bactericidal (2.92–3.73log_10_ decrease) for most concentrations against MT5094 while VAN log_10_ killing showed increased growth (1.52–3.09) except for the highest concentration tested (64 µg/mL, 1.44log_10_ killing). For FDXR28, IBZ was statistically better than FDX (*P* < 0.0001) and for MT5094, IBZ was statistically better than VAN (*P* < 0.0001).

**TABLE 2:** Time-kill kinetic data for IBZ, FDX, and VAN against *C. difficile* isolates^*a*^

		Drug concentration (µg/mL)					
Test strain	Drug	0	4	8	16	32	64
CD630	IBZ	.	−2.22	−2.82	−2.22	−2.4	−2.75
	FDX	.	−3.52	−3.57	−3.57	−3.14	−4.01
	VAN	.	1.45	−0.08	−0.68	−1.72	−2.52
	Control	2.92	.	.	.	.	.
R20291	IBZ	.	−1.61	−1.94	−3.43	−3.94	−2.74
	FDX	.	−3.53	−4.25	−4.45	−1.92	−2.7
	VAN	.	2.53	−1.18	−2.61	−2.13	−2.1
	Control	2.27	.	.	.	.	.
FDXR28	IBZ	.	−0.68	−1.9	−0.98	−0.77	−1.04
	FDX	.	2.29	2.56	2.56	2.64	1.26
	Control	2.55	.	.	.	.	.
MT5094	IBZ	.	−3.6	−3.73	−2.92	−3.13	−3.55
	VAN	.	3.09	2.74	2.77	1.52	−1.44
	Control	2.72	.	.	.	.	.

^*a*^ Results represent the change in log_10_ CFU/mL relative to 0 h (initial concentration). CD630 and R20291 are laboratory *C. difficile* strains. FDXR28 is a fidaxomicin non-susceptible isolate, and MT5094 is a vancomycin non-susceptible isolate.

### Light microscopy

**Fig 2:**
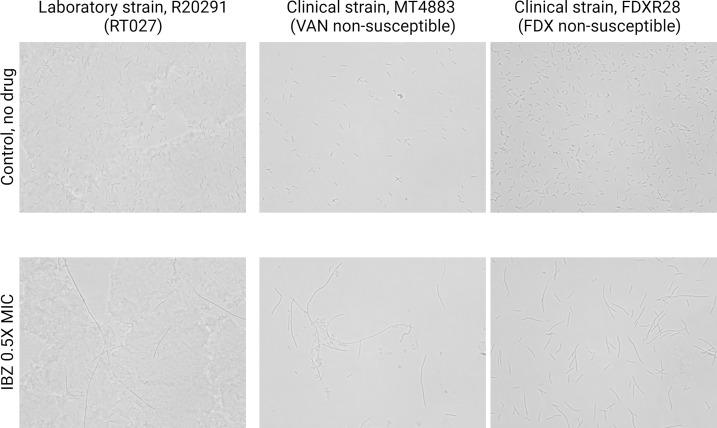
Representative light microscopy images demonstrating IBZ pharmacologic activity against *C. difficile* clinical strain MT4883, a multidrug-resistant strain with increased VAN (8 µg/mL), MTZ (2 µg/mL), and FDX (2 µg/mL) MICs and FDXR28 with reduced susceptibility to FDX (>16 µg/mL).

At IBZ 0.5× MIC, a similar increased cell length and filamentous phenotype were observed in isolates exposed to sub-MIC IBZ in reference strains and VAN/FDX reduced susceptibility isolates (Fig. 2).

## DISCUSSION

Antimicrobial resistance is important in the pathogenic spread of CDI including the most recent ribotype 027 epidemic. This epidemic was characterized by the emergence of two novel fluoroquinolone resistance genes at a time when fluoroquinolone antibiotics were commonly used (22). Recently, antimicrobial resistance has emerged to antibiotics that are commonly used to treat CDI, namely MET, VAN, and FDX (23). MET resistance emerged co-incident with the ribotype 027 epidemic possibly contributing to the increased virulence observed (5). Our group also demonstrated reduced clinical response rates in patients with MTZ non-susceptible strains (6). Although not as common or as well elucidated, antimicrobial resistance to *C. difficile* antibiotics is also emerging with VAN and FDX non-susceptible strains reported (3, 4). These results show that new antibiotic development in CDI must consider the emergence of antimicrobial resistance to antibiotics commonly used to treat CDI.

IBZ is a DNA polIIIC inhibitor with selective activity against *C. difficile*. In a phase IIa study, IBZ demonstrated a 100% clinical success rate in treating CDI with no recurrence (14). Phase III studies are being planned and a better knowledge of IBZ activity against MDR strains would allow for a better understanding of IBZ potential. Pharmacologic laboratory investigations allowed us to test the antibacterial activity of IBZ against comparator antibiotics (MET, VAN, and FDX) in our clinical strains with reduced susceptibility. Major findings include continued IBZ activity against multidrug non-susceptible strains using traditional MIC testing, an ATP-bioluminescence bactericidal assay, and time-kill assays. IBZ bacteriostatic or bactericidal activity was maintained for clinical strains with reduced susceptibility to VAN or FDX. These results expand and confirm the previous findings from Murray et al. that demonstrated similar MIC_50/90_ values (2/4 µg/mL) from 104 susceptible clinical strains and bactericidal activity against three clinical isolates (24). Like Murray et al., the activity of IBZ was significantly different than comparator antibiotics with MIC values for FDX being significantly lower than IBZ, VAN, or MET. It is worth noting that there was a wide spectrum of FDX MIC values potentially showing MIC creep with increased usage. Also, for the non-absorbable antibiotics such as IBZ, FDX, or VAN, further studies are needed to better understand how these MIC values relate to the concentrations in the gut. Also like Murray et al., IBZ exhibited bacteriostatic reduction from initial concentrations. With only two guideline-recommended antibiotics available for a common infection, the likelihood of resistance development is high. New antibiotic with a novel mechanism of action is needed. Taken together, these experiments provide strong *in vitro* evidence for the further development of IBZ as a treatment option for CDI including multidrug-resistant strains. The effect of IBZ against multidrug-resistant strains should be further explored in animal models to take full advantage of this unique mechanism of action.

In conclusion, this study demonstrated the potent bactericidal activity of IBZ against a large collection of *C. difficile* strains including multidrug-resistant strains. This study highlights the therapeutic potential of IBZ against multidrug-resistant strains of *C. difficile*.
